# Challenges in diabetes management with particular reference to India

**DOI:** 10.4103/0973-3930.54286

**Published:** 2009

**Authors:** Kavita Venkataraman, A. T. Kannan, Viswanathan Mohan

**Affiliations:** Department of Community Medicine, UCMS and GTB Hospital, Delhi, India; 1 Department of Madras Diabetes Research Foundation and Dr. Mohan's Diabetes Specialities Centre, WHO Collaborating Centre for Noncommunicable Diseases, Prevention and Control, Chennai, India

**Keywords:** Diabetes, diabetes management, non-communicable diseases, health care, India

## Abstract

Diabetes was estimated to be responsible for 109 thousand deaths, 1157 thousand years of life lost and for 2263 thousand disability adjusted life years (DALYs) in India during 2004. However, health systems have not matured to manage diabetes effectively. The limited studies available on diabetes care in India indicate that 50 to 60% of diabetic patients do not achieve the glycemic target of HbA1c below 7%. Awareness about and understanding of the disease is less than satisfactory among patients, leading to delayed recognition of complications. The cost of treatment, need for lifelong medication, coupled with limited availability of anti-diabetic medications in the public sector and cost in the private sector are important issues for treatment compliance. This article attempts to highlight the current constraints in the health system to effectively manage diabetes and the need for developing workable strategies for ensuring timely and appropriate management with extensive linkage and support for enhancing the availability of trained manpower, investigational facilities and drugs.

## Introduction

Non-communicable diseases were estimated to account for 35 million (60%) of the 58 million deaths globally in 2005. Of these, 72% were estimated to have occurred in low and lower middle income countries.[1] In India, 53% of all deaths in 2005 were estimated to be due to non-communicable diseases.[2] Non-communicable diseases pose a different and more complex threat to the health systems of countries, already faced with the unfinished agenda of infectious diseases, and maternal and child health problems. The hallmarks of these diseases namely long latency, chronicity, multi-organ involvement and need for long-term care make the management of chronic conditions difficult.

Type 2 diabetes mellitus exemplifies the management challenge in non-communicable diseases. Though recognized as a distinct clinical syndrome for centuries, our understanding of the disease, its causation, and mechanisms for progression are still evolving.[3] Over the past few decades, diagnostic criteria, and management algorithms for diabetes have seen rapid revisions. These are reflections of not just the translation of basic research into diabetes practice guidelines, but also an increased realization of the morbidity potential of the disease and its complications.

The global burden of diabetes was estimated to be 154 million in 2000, with a prevalence of 4.2% in the general adult population.[4] There were an estimated 37.76 million diabetics in India in 2004; 21.4 million in urban areas and 16.36 million in rural areas. Diabetes was estimated to be responsible for 109 thousand deaths, 1157 thousand years of life lost and for 2263 thousand disability-adjusted-life years (DALYs) during 2004.[5] The estimates for disease burden due to diabetes vary from 23 million in 2000[4] to 41 million in 2007,[6] the vast majority having type 2 diabetes mellitus. A substantial proportion of these patients will have diabetes-related complications. The percentages of patients having diabetic retinopathy, microalbuminuria and peripheral neuropathy in the Chennai Urban Rural Epidemiological Study (CURES) were 17.6, 26.9% and 26.1% respectively.[7–9] In the Chennai Urban Population Study (CUPS), 21.4% of diabetes patients had coronary artery disease, while 6.3% had peripheral vascular disease.[10,11] The health system needs to be geared to tackle these huge numbers, while ensuring health care that is universally accessible and of acceptable quality. This paper looks at the challenges diabetes poses to the health system globally and particularly in India.

### Global scenario

National guidelines and standards of care for diabetes are now available in many countries in the world. Despite this, the management of patients with diabetes in practice remains less than satisfactory in most countries. For example, nation-wide studies in UK and USA have shown that the prevalence of inadequate glycemic control (Glycosylated Hemoglobin (HbA1c) > 7%) in UK[12] is 76%, and 50% in the USA.[13] Data from the NHANES 1999-2002 in the US indicate that other components of diabetic control were also inadequate with only 39.6% patients having blood pressure values less than 130/80 mm Hg and 36% having LDL cholesterol below recommended levels (< 100 mg/dl).[13]

In Brazil, management goals set by the Brazilian Diabetes Society were achieved in 46% of the patients surveyed with respect to HbA1c, 24% for body mass index (BMI), 28.5% for systolic BP and 19.3% for diastolic BP.[14] In a cross-sectional study from 12 countries in Asia, 54% of those surveyed did not have a recorded value of HbA1c. The study measured HbA1c independently, and 55% were found to have values higher than 8%.[15] Studies in Thailand[16] and Pakistan[17] have also found that only 26.3% and 31.4% of patients achieved HbA1c less than 7%, respectively.

Barriers to effective diabetes management include both provider- and patient- related issues. Physician barriers include sub-optimal knowledge of guidelines, constraints of time and facilities, and attitudinal issues.[18] Providers are not always aware of the most effective interventions and tend to spend more time on ‘acute’ management than ‘chronic’ care.[19,20] The complexity of T2DM as a disease, and the multiple interventions required, make physicians wary of treating T2DM, especially since disease prognosis remains unpredictable in spite of aggressive management.[21]

Patients' lack of knowledge about diabetes care can impede their ability to manage their disease. This is important as better patient self-management ability is related to improved diabetes control.[22] Perceived quality of life is also lower in patients being managed aggressively, due to lack of dramatic disease-related symptoms and side-effects of interventions. This can affect patient compliance with medical advice.[23]

Various models of chronic care and interventions to improve control of diabetes have been tested for usefulness in different countries. Broadly, these health care models have focussed on reorganizing the health care services with better-designed delivery systems, providing support for improved self-management by patients, supporting physicians to take appropriate clinical decisions through guidelines and clinical information flows, and creation of linkages between the health care provider organization and other agencies that can support patient care.[24] Individual studies and meta-analyses have assessed such models in various settings and demonstrated that reorienting health systems to include some or all of the above lead to improved clinical, behavioural and diabetes knowledge outcomes in patients.[25–27] Specific interventions for provider education, patient education, financial incentives, feedback and reminders have also been found useful in some studies.[28]

Ethiopia has tested a model where nurses working in primary health centres have been trained to provide chronic disease care to patients. Nurse-led clinics are run once a week and specialist support is also made available once a month. Staff turnover and irregular supply of medicines were the limitations in implementation of the model.[29] A community-based intervention for nutrition and physical activity in rural Costa Rica was also found to be effective in improving glycemic control and weight loss within the 12 weeks of the study. Student nutritionists provided the nutrition interventions, while community volunteers undertook the physical activity interventions. Sustainability of the interventions and the effects could not be commented upon.[30] The Indonesian Endocrinology Society brought consensus guidelines for management of type 2 diabetes mellitus, which are now being followed by all health care professionals in Indonesia. Efforts are also being taken to increase the number of non-physician diabetes educators for patient education.[31]

### India

In India, limited studies have focussed on diabetes care and provide an insight into the current profile of patients and their management. In Diab-Care Asia, a multi-country study in Asia, the mean age of diagnosis among Indian respondents was 43.6 years. 50% had poor control as measured by HbA1c, and 54% had late severe complications.[32] In another pan-India study with patients recruited through providers, 70% of the patients were diagnosed by general practitioners. Only 43.4% patients had their BP checked at the time of diagnosis. The figures were 17.6%, 5.6% and 4.2% for eye examination, kidney function tests and lipid tests. In spite of these low percentages, 27.4% and 26.5% of those surveyed had elevated blood pressure and diminished vision at the time of diagnosis. Only 7-11% of patients had been tested for HbA1c, lipids, blood circulation and kidney function after diagnosis, and 47.2% monitored their condition only four or less times in a year.[33] Both studies cannot, however, be considered representative of diabetes patients in India due the lack of a defined population base and rigorous sampling.

Nagpal *et al.*, in a study among urban diabetics from middle and high income groups in Delhi, found that 41.8% of those tested had HbA1c greater than 8%, 63.2% had uncontrolled hypertension, and 74.5% had abnormal lipid profile. 79.4% were compliant with their medication, though 41.4% had not visited their health care provider in the past year. Only 13%, 16.2%, 32.1% and 3.1% of respondents had undergone HbA1c test, eye examination, serum cholesterol test, and foot examination, respectively, in the last year. Only 21.7% had heard of HbA1c or glycemic control.[34] Table 1 summarises the key findings from these studies.

**Table 1:** Summary of key findings – Indian studies on diabetes management

Parameter	Diabcare Asia[32]	CODI[33]	DEDICOM[34]
Year of study	1998	1999	2005
Total patients	2269	5516	819
Mean age (yrs)	53	54	54
% Type 2 diabetes	91	95	100
Mean HbA1c	8.9	Not measured	Not available
% having HbA1c < 7	50	Not measured	38
% tested for HbA1c	7.8	7.6	13
% tested for ophthalmic complications		35.1	16
% lipid tests		7.4	32
% having BP measured		54.3	Not available
% having serum triglycerides > 1.7 mmol/dl (150 mg/dl)	54		42

It is also probable that there is also substantial delay in diagnosis. In a study by Rayappa *et al*. in Bangalore, there was a ten-year difference in the age of diagnosis between the actively working and non-working respondents, a seven year gap between the highest educated and the least educated, and a four year lag between the highest and lowest socio-economic groups. Those with a late age at diagnosis also had multiple complications, implying delayed diagnosis of diabetes. Patients in this study also tended to evaluate diabetes control based on their perception of well-being. However, the mean blood glucose of those who felt ‘well’ was 180 mg/dL when last monitored.[35]

The awareness about the disease and its complications is also less than satisfactory among patients. Only 23% of self-reported diabetics, in a population based sample in Chennai, knew that diabetes could lead to foot problems, while only 5.8% knew that it could cause a heart attack.[36]

Compliance to medical advice, for a condition like diabetes, is an expensive affair, with the average cost per annum ranging from Rs 3000 to 10,000 in different studies.[37–39] In 2005, the median per annum cost for diabetes care was estimated to be Rs 10,000 for urban, and Rs 6,260 for rural patients.[39] Availability and affordability of anti-diabetic medication is another problematic aspect, in spite of “The National List of Essential Medicines” identifying glibenclamide, metformin, and insulin (soluble and lente) as anti-diabetic drugs that need to be available universally.[40] The availability of glibenclamide in public health facilities, for example, varied from 100 percent in Karnataka to 3.8 percent in West Bengal.[41] Given the uncertainty of availability of medicines, and the lack of pricing control over the private sector, compliance with medication becomes a serious issue. In one facility-based study, only 30% of patients reported to be compliant with medication, 37% with dietary advice, and 19% with exercise. Non-adherence was more in the lower socio-economic group.[42]

Provider issues are also very important for appropriate management. Inadequate knowledge, focus on acute management rather than preventive care, competing care demands and delay in clinical response to poor control are some of the physician-related issues in diabetes control in India.

## Discussion

Health care in India is provided by a variety of players, both governmental and non-governmental. The governmental system has a network of sub-centres, primary health centres and community health centres in rural areas, district hospitals, tertiary care hospitals and medical colleges in the cities. Though the system is based on the principle of state responsibility for free health care for the people without regard to their ability to pay, the focus continues to be on delivery of maternal and child health services.[42] Diabetes care is not explicitly part of the roles and responsibilities assigned to health personnel in the rural health care set-up, nor in pre and in-service training of personnel. Testing for blood glucose does not form part of the standard tests available in Primary Health Centres and Community Health Centres, and supplies for this test are not part of the central supply list.[43–46] Tertiary care hospitals bear the maximum load of patient care. The public health system tends to be under-utilised for all types of care, due to reasons of location, unreliable functioning of health facilities and increased indirect expenditures involved.[47]

The private sector covers a wide spectrum of providers, from the high-end corporate hospitals, charitable institutions, small nursing homes, sole practitioners, to unqualified providers. The private health sector operates in an unregulated market, and there are huge variations in the quality and type of care on offer.[47,48] Qualified practitioners tend to congregate in urban areas, while private providers in rural areas are likely to be less than fully qualified.[49,50]

The variety of health care providers, lack of national guidelines and protocols for health care services, including standards for health facilities, personnel and treatment protocols, makes it difficult to monitor and assure quality services across the board. Such differences have been noted in a range of services, including emergency medicine,[51] obstetrics[52] and paediatric care.[53] Health systems strengthening with development of nationally accepted management protocols for all levels of health care and appropriate monitoring for quality and accessibility are the foundation for improved health care across the board.

Care for non-communicable diseases, especially if it involves hospitalisation, is more expensive than care for acute illnesses. In urban Kerala, even non-poor families chose to utilize public hospitals for non-communicable disease hospitalisation, while preferring private facilities for acute illness-related hospitalisation in 1995-96.[54] While hospitalisation, surgery, medication and laboratory tests are the major drivers of cost, clinical practices driven by profit can substantially increase costs in the absence of well-defined management practices and clinical goal-setting.[55] Along with effective service delivery, innovative financing mechanisms will have to be developed to ensure risk pooling, and reduced financial burden on poor households.

The National Rural Health Mission[56] (NRHM) launched in 2005 and the new pilot National Programme for prevention and control of Diabetes, Cardiovascular diseases and Stroke[57] (NPDCS) offer opportunities for improving care for diabetes and other non-communicable diseases through service provision at the primary and secondary levels of care. Guidelines for the management of type 2 diabetes mellitus in the Indian context have also now been developed through a joint consultation by the Indian Council for Medical Research (ICMR) and WHO in 2005.[58] The matrix in Table 2 can be used for service delivery at various levels.

**Table 2:** Services for diabetes management

Activity	Community	Sub-centre	PHC	CHC	District Hospital
Health Education					
Identification of those at high risk					
Blood sugar testing					
Treatment initiation					
Management on insulin					
Screening for complications					
Follow-up for compliance					
Management of complications					

Modified from The National Commission on Macroeconomics and Health[59]

Effective management of people with diabetes is only a part of the solution for the problem of diabetes. Other aspects of care important from the perspective of diabetes control may be difficult to provide within the health system itself. Aspects related to the diet and amount of physical activity undertaken will be influenced by interplay of various sectoral policies and forces. Adherence to diet restrictions, for example, will depend on the sustained availability of inexpensive dietary substitutes in the market, their affordability and accessibility on a continuous basis to the patient apart from provision of appropriate dietary advice to the individual and the patient's motivation for adherence. These will require re-aligning of national or state policies for food procurement, pricing and marketing, to ensure lower prices, and improved access to healthy foods, and the opposite for those increasing health risk. Population-based strategies for health promotion and risk reduction, along with surveillance of trends in disease and risk factors are equally important components of any public health approach for diabetes control. The PACE project in Chennai has demonstrated the feasibility and the effectiveness of a large-scale multipronged diabetes awareness programme, and such approaches can complement effective diabetes management by increasing knowledge of diabetes and its prevention across the population.[60,61]

## Conclusions

Diabetes management remains a challenge for developed and developing countries alike. The implementation of evidence-based guidelines and restructuring of clinical care organization has yielded gains in some countries. There have been several attempts in developing countries as well to generate feasible and effective care systems. These initiatives and projects hold promise but much depends on the re-orientation of the overall health system for effective and sustainable care.

In India, as in other countries, the health system has traditionally been designed to cater to acute illness and maternal and child health concerns. The need for long-term care, for non-communicable diseases, is a relatively new health concern, and personnel and infrastructure are as yet not geared to face this task. The burgeoning load of diabetes is a real threat in India, underscored by the constraints of the health system in terms of manpower and capacity. Workable strategies for ensuring timely and appropriate management require extensive linkage and support for enhancing the availability of trained manpower, investigational facilities and drugs. Primary prevention through promotion of healthy lifestyles and risk reduction is recognized as the most cost-effective intervention in resource-poor settings. However, India will need to also plan for the care of the sizeable number of people with diabetes, in order to prevent and decrease morbidity due to complications. A health system strengthening approach with standards of care at all levels, nationally accepted management protocols and regulatory framework can help in tackling this challenge.
